# Hysteroscopic hysteroplasty for the treatment of T-shaped uteri in women with reproductive failure

**DOI:** 10.3389/fmed.2023.1269733

**Published:** 2023-12-22

**Authors:** Lu Ying, Zhang Li, Du Shangping, Zhou Tong, Li Liguo, Tong Qiaoli

**Affiliations:** ^1^Wenzhou Medical University, Wenzhou, China; ^2^Fuyang First People's Hospital, Fuyang, China; ^3^Yongkang Maternal and Child Health Care Hospital, Yongkang, China

**Keywords:** hysteroscopy, hysteroplasty, reproductive disorders, uterine malformation, treatment

## Abstract

**Objective:**

To evaluate the efficacy of hysteroscopic hysteroplasty in the treatment of uterine malformation complicated by primary reproductive disorders.

**Methods:**

Women with unexplained primary infertility, repeated *in vitro* fertilization (IVF) failure or repeated spontaneous abortion, and uterine malformations unrelated to diethylstilbestrol who visited the obstetrics and gynecology department of our hospital from January 2019 to December 2022 were included in the prospective cohort study. Uterine malformation in the patients was confirmed by three-dimensional ultrasound and diagnostic hysteroscopy. Hysteroscopic hysteroplasty was performed using a 5-mm diameter hysteroscope and 5-FR surgical scissors, and after 3 months, palliative care was proposed for patients with unexplained infertility or repeated spontaneous abortion, and after 6 months, IVF treatment was recommended for patients with repeated *in vitro* fertilization (IVF) failures, with a planned minimum follow-up time of 1 year.

**Results:**

A total of 83 women enrolled in the study, including 33 cases of primary infertility, 29 cases of repeated spontaneous abortion, and 21 cases of repeated IVF failure. No complications occurred during the hysteroscopic surgery. During the follow-up period, the clinical pregnancy rate of the women enrolled in the study increased to 77.1%, the live birth rate went up to 79.7%, the fetus delivered at full term accounted for 64.1%, and the cesarean section rate was 27.5%. The miscarriage rate was 9.4%.

**Conclusion:**

Hysteroscopic hysteroplasty can improve the reproductive outcomes in women with primary reproductive disorders and uterine malformations.

## Introduction

A rare disease of the Mullerian duct, T-shaped uterine malformation, occurs in an abnormal uterus (U1), characterized by abnormal uterine cavity shape and normal uterus contour (1). T-shaped uteri (U1a) are characterized by thickened lateral walls, hypoplastic cavity, two-thirds of the uterine body, and one-third of the cervix attached. Changes in uterine cervix as well as volume probably contribute to the reduction in the tolerance of endometrium, and this likely explains the increasing prevalence of uterine malformations in patients who suffer from recurrent abortion and infertility (2, 3). With regard to the T-shaped uteri, different research reports (4, 5) show that when the deformed uterus is not treated, the reproductive results are very poor. After a minimally invasive hysteroscopic hysteroplasty treatment was introduced, several studies have recorded the anatomical and/or reproductive results after the surgery, and the results are more effective (6, 7). However, there are limitations to these studies such as fewer samples, heterogeneity in the etiology of T-shaped malformations, retrospective design, and the different diagnostic and surgical techniques. This prospective cohort study was performed in women with repeated spontaneous abortion, primary infertility or repeated *in vitro* fertilization (IVF) failure, and T-shaped uterine malformation unrelated to DES (estradiol benzoate). These women were all treated surgically using standardized hysteroscopic techniques. The main objective of the research was to assess the impact of hysteroscopic hysteroplasty in cases of T-shaped uteri unrelated to DES on the rate of live birth. The secondary aim was to evaluate its effect on the rate of clinical pregnancy, rate of spontaneous abortion together with surgical, reproductive, and obstetric outcomes.

## Materials and methods

### Study population

Women with unexplained primary infertility, *in vitro* fertilization (IVF) or recurrent spontaneous abortion, as well as T-type uterine malformation unrelated to diethylstilbestrol (DES) who were treated in the gynecology and obstetrics department of our hospital from January 2017 to December 2018 were taken as the study population for a prospective cohort study. The follow-up period after hysteroscopy and hysteroplasty was at least 1 year (until December 2020).

#### Inclusive criteria

Women who have not given birth with suspected uterine anomalies include those with a medical history of unexplained primary infertility, recurrent spontaneous miscarriage (RSM), or repeated *in vitro* fertilization failure (RIF).

#### Exclusive criteria

Body mass index (BMI) >30 kg/m^2^; past history of term pregnancy; pregnant; malignant tumor; systemic severe chronic disease; infertility-related gynecological conditions, such as hysteromyoma as well as endometriosis; suspected acquired T-shaped uteri (Asherman syndrome diagnosed by hysteroscopy); suspected or confirmed intrauterine DES exposure; and previous hysteroscopic surgery.

The primary T-shaped uteri was diagnosed in all the women by 3D ultrasound and hysteroscopy. The same gynecologist who was responsible for the 3D ultrasound examination of the abdomen and vagina during the luteal phase (days 21–26) also performed the diagnostic hysteroscopy. The ethical approval for this study was obtained from the hospital's ethics committee with all patients signing an informed consent form.

## Method

During the first phrase of the menstrual cycle (days 6–10), hysteroscopic hysteroplasty was performed after menstruation stopped. In the state of conscious sedation, we used a diameter of 5 mm hysteroscope for colposcopes, a 30 degrees anterior oblique mirror as well as a 5-FR operation thoroughfare and salt solution (sodium chloride 0.9%) as the expansion agent. Electronic flushing as well as suction system (Endomat; Karl Storz, Germany) was used to provide a steady pressure *in utero* of 50 mmHg by tuning the flushing pressure to 100 mmHg, the suction pressure to 0.2 bar, and the flow rate to 240–370 mmHg. Each surgery was performed by the same endoscopic specialist.

Hysteroscopic hysteroplasty uses 5-FR surgical scissors to make two lateral incisions on the lateral wall of the uterus to avoid electrosurgical operation. Under the naked eye, from the isthmus to the fundus, the myometrium is cut through on both sides perpendicular to the lateral wall of the uterus. In order to obtain good results, the cut should pass through the same groove several times in advance, and the maximum cut depth is 7 mm to avoid perforation. After the surgery, a silicone film or gel was not implanted in the uterus for mitigating the danger of failing to achieve a clinical pregnancy after 12 months of regular, unprotected sex. All patients were treated with sequential estrogen and progesterone for 3 months to avoid pregnancy.

### Observation parameters and follow-up

At 3 months follow-up, the cases of natural pregnancy and *in vitro* fertilization were recorded from medical history and entered into the database. The patients with unexplained couples' infertility (RSM) were treated in a palliative mode for natural pregnancy for 6 months and *in vitro* fertilization (IVF) after 6 months. In the case of palliative treatment, follow-up was conducted every 2 months with planned gynecological follow-up at 6 months. The definition of the clinical pregnancy is ultrasound diagnosis of intrauterine pregnancy. The definition of abortion is spontaneous abortion before 24 weeks of pregnancy and live birth is a live fetus delivered after 24 weeks of gestation. Follow-up was done 1 year after hysteroscopy.

### Statistical analysis

Correlation analysis was done using SPSS 24.0 software (IBM Corporation, Armonk, New York). Values are expressed as mean±standard deviation (SD) for continuous variables while the categorical variables are represented as proportions, which were analyzed using Fisher's exact test. The *post-hoc* analyses were used where appropriate and were then deemed to be statistically valid if the *P* < 0.05.

## Results

### Demographic data

In total, the study comprised 83 women who had not given birth. T-shaped uteri was confirmed by hysteroscopy and 3D ultrasound in all patients. There was no intrauterine DES exposure or acquired malformation. The general statistical data of the selected cases and the indications of hysteroplasty are shown in Table 1. The hysteroscopic hysteroplasty procedure was successfully completed in all patients without obvious complications. The average operation time was 11.2 ± 4.2 min.

**Table 1:** Baseline characteristics of included population.

Age	36.2 ± 2.83
BMI (kg/m^2^)	24.3 ± 2.29
Exposure history of estradiol benzoate (*n*, %)	0 (0%)
History of oral estrogen contraceptives (*n*, %)	46 (68.7%)
Operation indication (*n*, %)	Primary infertility 33 (39.8%)
	Recurrent spontaneous abortion 29 (34.9%)
	Repeated *in vitro* fertilization failure 21 (25.3%)

### Follow-up and pregnancy

The patients were followed up for 12–34 months after surgery, 14 months on average. Of the 83 women, 19 were not pregnant after surgery, four were diagnosed with colon cancer after hysteroscopy, three cases of peritonitis were observed as a result of diverticulitis, one refused to undergo IVF again due to psychological problems, 11 were unwilling for pregnancy on their own will, and 64 were pregnant. The overall clinical pregnancy rate after hysteroscopy was 77.1% (64/83).

Among the 64 pregnant women, 51 gave birth to live fetuses, with a live birth rate of 79.7% (51/64), which increased from 0% before the surgery to 79.7% after the surgery (*P* < 0.001). Among the 51 women, 11 underwent cesarean section, accounting for 21.6%. The remaining 13 women delivered through clinical pregnancy, seven women through continued pregnancy, and six cases of miscarriage were also observed. The spontaneous abortion rate was 9.4%, and the rate of miscarriage reduced from 100% before the pregnancy to 9.4% after the surgery (*P* < 0.001). Pregnancy complications associated with uterine surgery such as cervical insufficiency, adherent placenta, uterine rupture, or any others were not observed in pregnant women.

Among the pregnant women, there were 33 cases of primary infertility and 25 cases of pregnancy, with clinical pregnancy rate of 75.8% (25/33), 19 cases of live fetus (76.0%, 19/25), three cases of spontaneous abortion (12.0%, 3/25), and three cases of continuous pregnancy (13, 24 and 34 weeks of pregnancy). Nine cases out of 25 cases of clinical pregnancy (36.0%) were naturally pregnant within 3 to 9 months after hysteroscopy and 19 women received IVF (one time of egg donation), with a clinical pregnancy rate of 78.9% (15/19) and median of one time of IVF (one to three times).

Among the 29 cases of RSM, 23 cases were pregnant after hysteroplasty, with a clinical pregnancy rate of 79.3% (*n* = 23/29). Of them, 15 pregnant women gave birth to live fetuses, and the live birth rate was 82.6% (19/23), 0% before and 82.6% after hysteroplasty (*P* < 0.001). Four cases of spontaneous abortion (17.4%, *n* = 4/23) and two cases of continuous pregnancy (7 and 12 weeks of pregnancy) were reported. Miscarriage rate dropped from 100% before pregnancy to 17.4% after hysteroscopy (*P* < 0.001). Of the 23 cases of clinical pregnancy, 9 cases (39.1%) became pregnant naturally within 3–9 months after hysteroscopy, and 14 cases received IVF (three times of egg donation). The clinical pregnancy rate was 85.7% (12/14), and the median was one time of IVF (1–3 times).

Of the 21 women with RIF, 16 were pregnant after hysteroscopy, with a clinical pregnancy rate of 76.2%. Of these, there were 13 cases of live birth, 81.3% (13/16), one case of spontaneous abortion (9.1%; *n* = 1/11), and two cases of continuous pregnancy (12 and 23 weeks of pregnancy). Two women (12.5%) out of 16 women opting for clinical pregnancy were naturally pregnant during follow-up after hysteroscopy, and 14 women through IVF (three times of egg donation), and the clinical pregnancy rate was 78.6% (11/14), The median is one IVF (1–3 cycles).

## Discussion

**Figure 1:**
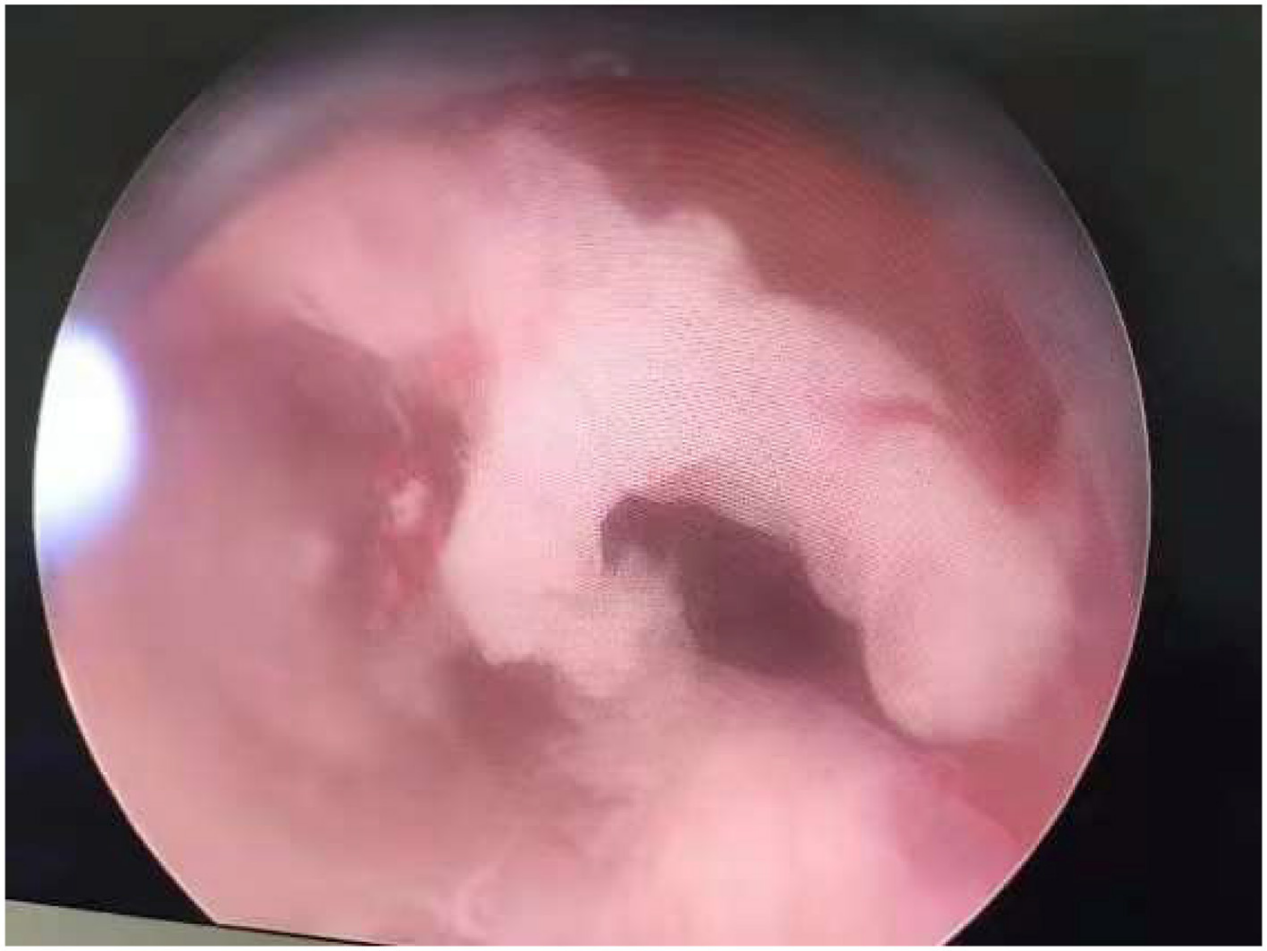
Typical T-shaped uteri of a 29-year-old woman.

**Figure 2:**
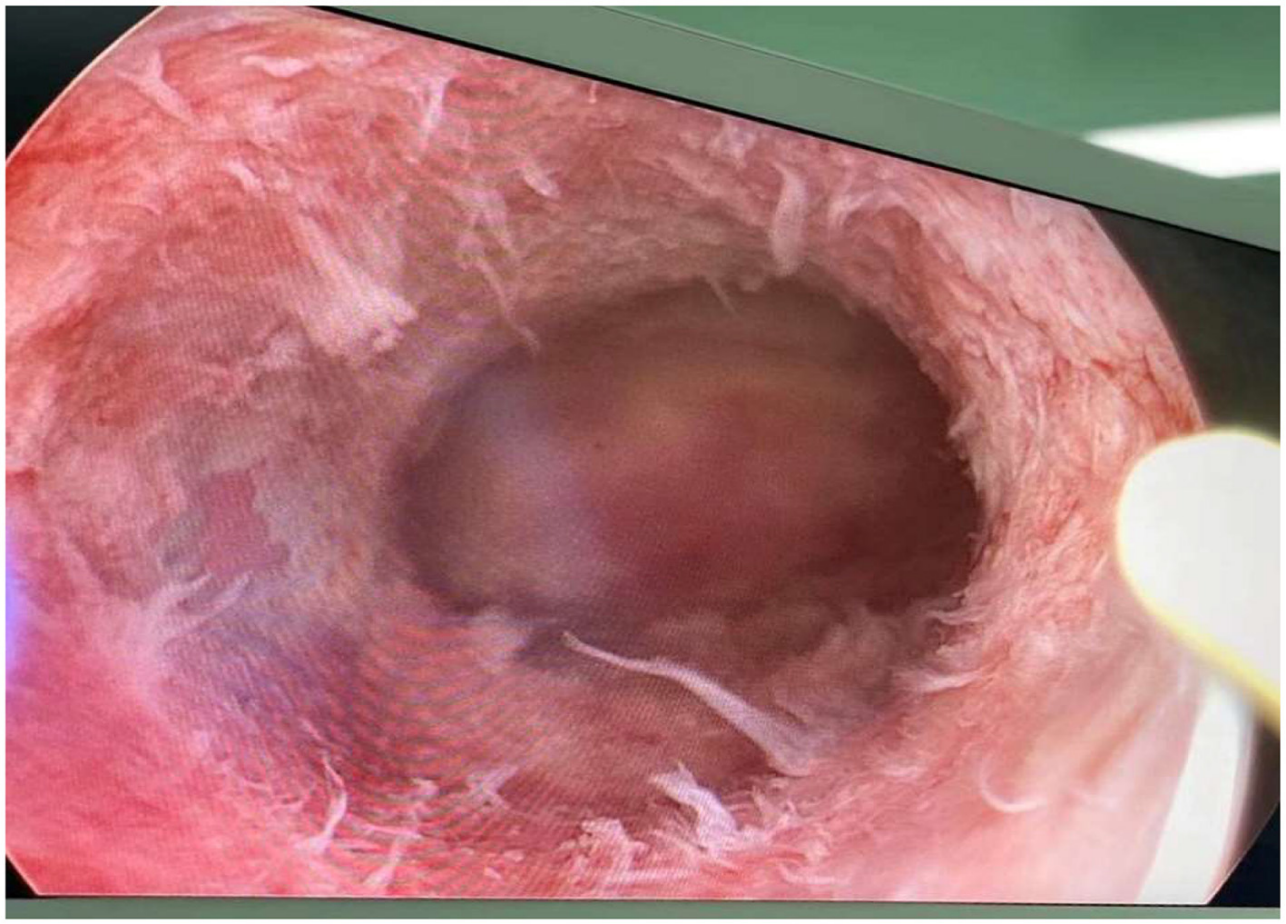
Typical T-shaped uteri of a 32-year-old woman.

Among the patients in this study, complications of pregnancy associated with uterine surgery have not been recorded. The incidence of full-term delivery is 64.1% and the cesarean section deliveries are 27.5% (14/51). The clinical pregnancy rate is 77.1%, which is almost equivalent to the rate of live birth (79.7%) and the rate of abortion is 9.4%. Overall, the results of this study confirm that endoscopic hysteroplasty can improve the reproductive outcome in women who have T-shaped uteri and primary reproductive disorders (please see Figures 1, 2 and Table 2).

**Table 2:** Comparison of pregnancy status of patients.

	**Total (*N =* 83)**	**Primary infertility (*N =* 33)**	**Repeated *in vitro* fertilization failure (*N =* 21)**	**Recurrent spontaneous abortion (*N =* 29)**	**P**
Age	36.2 ± 2.83	35.9 ± 2.71	36.2 ± 2.96	36.9 ± 2.89	0.735
Clinical pregnancy	64 (77.1%)	25 (75.8%)	16 (76.2%)	23 (79.3%)	0.629
Live births	51 (79.7%)	19 (76.0%)	13 (81.5%)	19 (82.6%)	0.372
Term pregnancy	41 (64.1%)	17 (68.0%)	9 (69.2%)	15 (65.2%)	0.295
Natural abortion	6 (9.4%)	3 (10.3%)	1 (7.7%)	2 (10.5%)	0.378
Ongoing pregnancy	7 (10.9%)	3 (10.3%)	2 (15.4%)	2 (10.5%)	0.413
Clinical pregnancy of natural pregnancy	9 (14.1%)	3 (12.0%)	2 (12.5%)	4 (17.4%)	0.216
Live labor after natural pregnancy	7 (77.8%)	2 (66.7%)	1 (50.0%)	3 (75.0%)	0.013
RVF conception	47 (73.4%)	19 (76.0%)	14 (87.5%)	14 (60.9%)	0.009
Clinical pregnancy after RVF	38 (80.9%)	15 (78.9%)	12 (85.7%)	11 (78.6%)	0.718
RVF live pregnancy	33 (86.8%)	13 (86.7%)	11 (91.7%)	9 (81.8%)	0.624

Improvement in reproductive outcome is probably related to the remodeling of uterine morphology, enhanced compliance of uterus, improved uterine vascularization, and endometrial receptivity (8, 9). However, considering many mechanisms related to endometrial receptivity, such as immune factors, the determination of the precise mechanism has not been accomplished. On this basis, the impact of hysteroscopy on the integrity and function of the uterus during subsequent pregnancy seems to be limited. Similar to other studies (10), in terms of delivery, there were no pregnancy complications relevant to uterine surgery that have been recorded. Full-term births occurred in 64.1% of cases and the cesarean section rate was 27.5% (14/51), lower than previous studies. Theoretically (11), vaginal delivery is not a contraindication, although hysteroscopic hysteroplasty may image the fragility of the uterus and increase the danger of uterine rupture in theory. However, in previous studies (12), the ruptured uterus was recorded in only one case at 26 weeks of pregnancy (not during labor), and this is a risk that can therefore be regarded as extremely low.

About 30 years ago, a ban was imposed on the use of DES, which is the reason why intrauterine exposure to DES in young infertility patients nowadays predisposes them to congenital T uterine malformations. On this basis, the effect of hysteroscopic hysteroplasty in patients with uterine anomalies not exposed to DES was studied in two recent prospective studies and a retrospective research (13, 14). Retrospective analyses of the reproductive outcomes of 62 patients who had T-shaped uteri were conducted by Alonso Pacheco et al. (13), who reported a clinical pregnancy rate of 59.7% and a postoperative live birth rate of 75.6%, and the study involved lateral hysteroplasty using conventional 26-FR electrosurgery. Thirty patients with uterine malformations, of which 12 were T-shaped uteries, were prospectively evaluated by Di Spiezio Sardo et al. (14) using the hysteroscopic outpatient hysteroplasty for the enlargement of malformed uterus (Home-DU). In this case, the live rate of the patients who had primary infertility and recurrent spontaneous miscarriage was up to 71%. In their prospective research of 56 women who had primary infertility, primary T-shaped uteri, recurrent miscarriages, or *in vitro* fertilization failure, Ludwin et al. (15) performed hysteroscopic hysteroplasty, which was carried out under conscious sedation with the diameter of 5 mm hysteroscope of bipolar forceps system by the colposcope. In this study, the rate of clinical pregnancy (77.1%) and the rate of live birth (79.7%) were markedly improved, and the spontaneous abortion rate (9.4%) was decreased, which was consistent with the results reported previously (16). The results of this study confirmed the effectiveness of hysteroscopic hysteroplasty. The reason why it is superior to previous studies may be the forward-looking design as well as the rigorous hysteroscopy-based diagnostic criteria, which exemplify the gold standard for diagnosis and allow the unambiguous identification of abnormalities. In addition, the selection to exclude T-shaped uteri associated with DES minimized study error, conforming to clinical practice.

In conclusion, data on women who failed to give birth due to T-shaped uteri are provided in this study. They were diagnosed and treated by normative methods surgically. T-shaped uteri was identified as the sole potential known factor for infertility and a set of strict inclusive criteria was followed to enroll participants in the study. The strict inclusion criteria allowed for the identification. The results confirmed that endoscopic hysteroplasty can improve the reproductive outcome in women who had T-shaped uteri and primary reproductive disorders. Comparing this study's results and previous ones, hysteroscopic hysteroplasty combined with colposcopy as well as sedation appears to be an effective way of improving reproductive outcomes with no obvious obstetric complications. However, this study also has certain limitations, such as the lack of a control group and limited sample size.

## Data availability statement

The raw data supporting the conclusions of this article will be made available by the authors, without undue reservation.

## Ethics statement

The studies involving humans were approved by the Ethics Committee of Fuyang First People's Hospital. The studies were conducted in accordance with the local legislation and institutional requirements. The participants provided their written informed consent to participate in this study.

## Author contributions

LY: Writing – review & editing, Investigation. ZL: Methodology, Writing – original draft. DS: Methodology, Writing – original draft. ZT: Methodology, Writing – original draft. LL: Methodology, Writing – review & editing. TQ: Data curation, Writing – review & editing.
